# Sacred whores and fire mamas: hybrid femininity and masculinity in Michigan’s Lakes of Fire regional burn

**DOI:** 10.3389/fsoc.2026.1683309

**Published:** 2026-06-19

**Authors:** Natalie J. Adamyk

**Affiliations:** Department of Sociology, University of Toronto, Toronto, ON, Canada

**Keywords:** Burning Man, cultural events, dominant femininity, dominant masculinity, emotional labour, hybrid femininity, hybrid masculinity, Lakes of Fire

## Abstract

This article explores the gendered emotional labour done by participants or “Burners” at Lakes of Fire, an annual regional Burning Man event held in Michigan. This paper conceptualizes Burners as embracing culturally dominant forms of hybrid masculinity and femininity, which also structure their emotional labour. Drawing on 42 one-on-one semi-structured interviews with male and female-identifying participants, I respond to calls from scholars to produce more relational analyses of gender within cultural studies scholarships. Results from the interviews demonstrate that, in taking on roles as empowered maternal figures and sexually progressive caretakers, the women embraced progressive but maternal communal mothering roles which require intensive feminine emotional labour. These “Burner femininities” enabled them to carry out the emotional labour required to sustain Lakes communally. In contrast, male Burners constructed hybrid forms of Burner masculinity by rejecting toxic and hyper masculinity while embracing more progressive masculine role and identities. However, men also embodied more leadership and managerial roles when undertaking emotional labour, in contrast to the explicitly maternal roles held by women. Burner masculinity and femininity therefore act relationally, by mutually reinforcing certain types of emotional labour as the largely separate domains of women and men. This paper also discusses the ways in which cultural events such as Lakes of Fire hold the possibility to both subvert and replicate more traditional understandings of gender.

## 1 Introduction: burns and masculinity

One of the most successful cultural events in the world, Burning Man is an art-centered camping event taking place annually in the summer. Along with smaller counterpart “Burns” held throughout the U.S. and the world, the original “Big Burn,” located in Black Rock City, Nevada, is now attended by up to 87,000 participants annually (U.S. Bureau of Land Management, 2025). Burning Man gained its name from the tradition that takes place on each Burn’s final night: the Burning of the effigy, where an elaborate wooden sculpture is burned to honour the dead (Shister, 2019, p. 5; see also St John and Gauthier, 2015). Ever since 1986 when the original Burn was held by founders Larry Harvey, John Law and Jerry James on a San Francisco beach, the larger Burning Man community, known collectively as “Burners,” have been notable for their commitment to the creation of decommodified, self-sustaining communities (Shister, 2019).

One of the larger offshoot Burns that embraces these values is Lakes of Fire in the northern Midwest. Colloquially known as “Lakes” and located on campgrounds off of Lakes Michigan, it becomes the temporary home to approximately 3,500 camping Burners annually in late June over the span of a week (Lakes of Fire, 2021). At Lakes, as with all Burns, the 10 Burner Principles are central to how both community and labour are structured. To establish a self-sustaining community, Burning Man’s original creators took many of the ritualistic and communal aspects emblazoned in the major religions and situated them within a secular space (Shister, 2019). The Ten Burner Principles thus resemble the Judeo-Christian Ten Commandments and underpin Burns’ social organization, providing Burners with “a guide for how to relate to one another in the pursuit of shared goals” (Lakes of Fire, 2021, official website).

First of these are the “foundational Principles” which “presuppose productive and engaged activities” with the goal of “creating shared experiences within the community” (Radziwill and Benton, 2013, p. 7). These Principles include *civic responsibility; immediacy; gifting; decommodification*; and *participation*. In contrast, Lakes of Fire’s “operational Principles” are rooted in both radical acceptance and an expansive sense of community. They include: *radical inclusion*; *radical self-expression; communal effort; leave no trace* (or commitment to environmental stewardship); and *radical self-reliance.* Both types of Principles also imply significant emotional labour. For example, gifting specifies that “labour, intellectual contributions, emotional and moral support” should be freely given by Radziwill and Benton (2013, p. 9; see also St John and Gauthier, 2015). Meanwhile, the operational Principles necessitate acceptance of others (Radical Inclusion), being comfortable with personal expression (Radical Self-Expression), and communal-mindedness (Communal Effort) (2013, p. 10). These Principles also emblemize progressive ideals related to aspects such as sexual consent and decommodified living (p. 11). Burners are also expected to both instruct newcomers to Lakes on these topics and provide a listening ear to their fellow festival goers (Chen, 2012). Emotional labour is thus intrinsic not only to the production and maintenance of art and community at Lakes, but in binding Burners together in a community through shared values and obligations (Turner, 2009; Shister, 2019).

As a demonitized cultural event which requires ongoing outpourings of emotional labour, Lakes of Fire successfully engages thousands of volunteers who maintain a vibrant and welcoming community (see Kaiser and Green, 2008, 2011). This paper therefore focuses on how Burners adopt culturally-prescribed gender identities in undertaking this required emotional labour. Drawing from 42 interviews with 21 male and 21 female-identifying Burners, I ask the following questions: How do participants create and embody unique forms of masculinity and femininity within the Lakes of Fire space? How do these “Burner” masculinities and femininities function relationally, and how does this impact how emotional labour is distributed among participants? And, how do these relations present opportunities for Burners to both subvert and reinforce traditional understandings of gender and community? Drawing on Connell’s typology of relational masculinities and femininities (Connell, 1995; Connell and Messerschmidt, 2005), and relevant gender events literature (Pielichaty, 2015; Finkel and Platt, 2020; Coyle and Platt, 2018; Grabher, 2021), I explore how these gendered relations serve as mechanisms through which Burner understand and engage in emotional labour.

This paper represents an important contribution to cultural events scholarship for several reasons. First, it responds to calls by scholars to analyze cultural events not specifically devoted to gender and sexuality, such as Burns, which still constitute dynamic sites for the production of unique gender identities and relations (see Grabher, 2021). This is significant insofar as DIY cultural events such as regional Burns are often important sites for both the subversion and recreation of gender structures and relations (Kaiser and Green, 2008). Burns such as Lakes of Fire also provide important spaces for marginalized individuals from, for example, the LGBTQ+ and polyamorous communities to supplement or even replace their own families and communities from the “default” or outside non-Burner world (Kaiser and Green, 2011). This paper therefore provides valuable analysis of how Burns constitute important spaces for reimaging communal spaces and gender roles.

Second, this research frames gender identities as being relational in nature, and therefore depicts masculinity and femininity as mutually necessary in directing the emotional labour needed to sustain cultural events such as Burns. In doing so, this paper respond to calls from gender scholars like Connell, who originally conceptualized masculinity as existing in asymmetrical relation to femininity (1995, p. 65; see also Connell and Messerschmidt, 2005). However, since the introduction of Connell’s typology, the subsequent research on gender and masculinity has not meaningfully explored the mutually-reinforcing nature of masculinity and femininity in these spaces (p. 66). This focus overlooks the fact that Burners play essential roles in constructing masculinity and femininity relationally, as wives, husbands, mothers, and fathers, which they bring to their roles within cultural events (Connell and Messerschmidt, 2005, p. 34). These identities also intertwine with cultural standards of masculinity and femininity, ultimately shaping how individuals perform emotional labour. In exploring how Burners create relational, culturally-specific gender identities, this paper provides a valuable contribution to existing gender and cultural events research.

In this article, I first provide an overview of the history and culture of Lakes of Fire and Burning Man more generally. I then discuss the methods used for participant recruitment, interviews, and coding. Next, I discuss Hochschild’s emotional labour and transmutation concepts and review the research on emotional labour within cultural events, before discussing relational approaches to gender, and masculinity and femininity. I then go over the findings from the interview data before reviewing them in the Discussion section. I conclude the paper by discussing limitations and provide future research directions.

## 2 Lakes of Fire and Burning Man: an overview

Lakes of Fire originally began in 2009, like its larger counterpart in Nevada, as a DIY event devoted to the creation of participant-driven art within a decommodified space (Lakes of Fire, 2021, official website). Both aims are clearly outlined in the Mission Statement on the Lakes of Fire’s website home page:

Welcome! Lakes of Fire is an annual collaborative celebration of art and community, steeped in friendship, fire, and wonder. This event will be created for you, and by you – your creativity, art, and relentless enthusiasm are what makes it happen. Without you, there is no home to return to!

Our home hosts theme camps and open camping, but everywhere you look there will be music, art, and fiery fun. Lakes of Fire revels in the creative carnival atmosphere and non-stop smiles you have always brought to it.

Lakes of Fire is the Great Lakes Region’s official Burning Man Regional Event and conducted in accordance with the 10 Principles (Lakes of Fire, 2021).

Burns like Lakes require the extensive efforts of participants, who contribute their time and unpaid labour in capacities such as camp set-up, the creation of art displays, and running or helping with Lakes’ many theme camps. To accomplish these tasks, Burners take on both formal and informal leadership positions, including as camp leaders who are responsible for feeding and even administering medical attention to other Burners. Some also work as rangers who are responsible for securing the safety of the Lake Michigan campgrounds (Lakes of Fire, 2021). More broadly, experienced “veteran” Burners are also expected to “pass down” the Principles to new Burners, ingraining in them the values necessary to become good Burner citizens (Chen, 2012, p. 55). Additionally, Lakes’ emphasis on consent, often referred to as the unofficial “eleventh Principle,” requires Burners to teach and counsel others around relationships and sexual encounters (Lakes of Fire, 2021). Lakes, like other Burns, simultaneously emphasizes communitarianism and self-reliance, which are also enshrined within the Principles.

According to Shister (2019), Burning Man founder Larry Harvey enshrined the Ten Principles to reconcile the tendency among Burners to champion “individual autonomy” over the “institutional resilience” the founders were seeking (p. 22). Despite borrowing many ritualistic elements from existing organized religions, the original Burning Man stressed personal agency within a postmodern landscape. Burner culture also embraces personal experience as the only true means of knowledge and authority, as “herein resides the supreme tenet, [that] people are capable of creating anything” (Shister, 2019; p. 43). As cultural events, Burns defy established norms and practices while simultaneously ingraining new ones to create a larger culture of self-reliance. This ensures that Burners form communities which maintain a shared vision and act cohesively (Chen, 2009, p. 51), and as a result, “pass down” Principles to new Burners, to ingrain the values necessary for them become good “Burner citizens” (Chen, 2012, p. 79).

While the literature on Burning Man provides valuable analysis of Burns as unique cultural fields with a vibrant art culture and history (Chen, 2009; Clupper, 2007; St John, 2019, 2020; Turner, 2009), it largely overlooks the explanatory power of emotional labour in understanding how Burners create these community-centered festivals. Additionally, as St John and Gauthier (2015) note, there are few scholarly accounts of “the translation, modification and adaptation of the Ten Principles” beyond those which focus on Nevada’s “Big Burn.” Indeed, while over 50 different Burns worldwide have adopted the Ten Principles in recent decades, these events themselves “are not straight-forward exports or simulacra,” but regionally-influenced interpretations of the Burning Man “operating formula” (2015; see also Radziwill and Benton, 2013; Wachs, 2014). In focusing on one of the U.S.’s largest affiliate Burns, this article highlights how regional differences allow participants to create communities (and gender relations) in unique ways which are influenced by the surrounding culture, while still upholding the founders’ vision of a self-sustaining community, using emotional labour as a conceptual lens.

## 3 Emotional labour in cultural events

A term popularized by sociologist Hochschild (1983), emotional labour refers to the psychological and physiological changes workers make to fulfill their organization’s emotional and interpersonal requirements, such as through interactions with customers (p. 15). Hochschild also emphasized emotional labour’s gendered nature, with women disproportionately filling caretaking and nurturing roles and positions. In contrast, men’s emotional labour more commonly involves the enforcement of rules and intimidation of others, such as in the role of tax collectors (p. 43; see also Polletta and Tufail, 2016). Hochschild also conceptualized “transmutation,” the process through which workers’ emotional labour essentially becomes owned by their employers in their interactions with others, including customers and patients (Hochschild, 1983; see also Bolton, 2003; Brook, 2009). Their emotional labour is thus transmutated on the company’s behalf, as workers come to identify with their employers’ goals, using “deep acting” to convey genuine emotions in their jobs (1983, p. 31; see also Brook, p. 533). However, emotional labour also encompasses not only “the preserve of frontline-line service workers,” but is “utilized in varying degrees by all workers in multiple areas of organization” (Brook, 2009; p. 538).

In analyzing emotional labour within demonitized cultural events, Hochschild’s transmutation concept characterizes individuals’ motivations as not merely financially driven but stemming from workers’ deep identification with their organizations’ goals and mission statements (1993, p. xii; see also Brook, 2009, p. 533). Emotional labour scholars have primarily explored paid work in multiple fields and sectors, including law, nursing, healthcare and teaching (see Mann, 2007; Harris, 2010; Smith, 2012; Guy et al., 2015; Riley and Weiss, 2016). However, Hochschild provides an important lens for conceptualizing Burners’ labour, which likely involves transmutation in ways similar to those of paid employees.

Existing cultural events literature has explored emotional labour within festival and decommodified spaces, focusing on the centrality of emotional labour to the creation of events, and its gendered nature (Hardt and Negri, 2000; Flowers and Swan, 2015; Prakash, 2019; Grabher, 2021; McGillivray and Walters, 2024). For example, Hesmondhalgh and Baker (2008) conceptualize work within cultural events as labour which produces intangible goods and services, including cultural products and knowledge, which are more likely to be undertaken by women and feminine-presenting people (p. 97–99). Williams (2023) similarly characterizes “cultural labour” as constituting a wide range of roles, including “manual, cognitive, emotional and affective” forms of work, all of which are intertwined (2023, p. 460). This labour usually involves ongoing collaboration among the cultural field’s participants which, in the case of visual and performance art, often produces strong emotions in colleagues and audiences (Hardt and Negri, 2000; Williams, 2023, p. 461). Williams (2023) also notes how volunteers in high-end London theatres use collaborative emotional labour to respond to the needs of visitors in the organization, while also providing a supportive community for colleagues (p. 462). This is likely similar to how Burners engage in emotional labour to create cohesive communities (Chen, 2009) through gendered dynamics which are relational in nature.

## 4 Hegemonic and dominant masculinities

According to Connell (1995), gender identities work through the creation of culturally-specific “idealistic examples of masculinity [and femininity]” (1995, p. 42; see also Connell and Messerschmidt, 2005). Gender is also formed in tandem with larger social structures and cultural practices, and masculinity therefore cannot exist except in relation – and contrast – to femininity, and vice versa (1995, p. 42). All gender hierarchies are also shaped by patriarchy, in which men hold power while women are excluded from doing so to varying degrees. Patriarchy encompasses political, financial and religious systems and configurations, which also vary historically across countries and cultures (Facio, 2019). Additionally, Connell frames gender as existing within hierarchies which organize different forms of masculinity and femininity. Of these, *hegemonic masculinity* constitutes the most culturally dominant and exalted form and embodies the “currently accepted answer to the problem of the legitimacy of patriarchy,” which maintains men’s dominance and, in relation, women’s subordination (1995, p. 76). Hegemonic masculinity is also achieved through what Messerschmidt (2018) refers to as “discursive persuasion,” where most people in a society “consent to and embody unequal gender relations between men and women, masculinity and femininity, and among masculinities” (p. 86). Hegemonic masculinity thus serves as a structural and symbolic ideal through which elite men are able to maintain cultural dominance, both over women and subordinate groups of men.

Related to hegemonic masculinity is *toxic masculinity,* often used colloquially to refer to any type of abusive or otherwise undesirable “masculine” behaviour (Blair and Hoskin, 2022). While hegemonic masculinity often contains many of the characteristics emblematic to toxic masculinity, such as the oppression of women (2022, p. 2005–2007; see also Connell, 1987), the latter is largely a colloquial definition, while hegemonic masculinity is always meant to be understood hierarchically, as it shapes all other masculinities and femininities (Messerschmidt, 2018). Within this paper, “toxic masculinity,” when it is used, therefore refers to a popular definition rather than a hierarchical one.

In contrast, *dominant masculinity* refers to culturally-ascendent forms of masculinity which, unlike hegemonic masculinity, do not reinforce men’s material or cultural dominance over women and other subordinate men. Messerschmidt (2020b) coined the term in response to critiques that certain forms of masculinity which scholars commonly referred to as “hegemonic” were not actually instrumental in advancing men’s power and dominance over women (p. 98; see also Beasley, 2008). Dominant masculinities and femininities therefore serve as cultural standard bearers within a given society, but do not reinforce men’s material or cultural dominance on a hierarchal basis as hegemonic masculinity does (Messerschmidt, 2020b, p. 25; see also Messerschmidt, 2018). For example, within more liberal cultures and spaces, forms of masculinity which emphasize egalitarianism between genders and men’s adoption of feminist views may be the most culturally embraced, and therefore function as the “dominant” masculinities (see Cornell, 2009). However, they do not reinforce men’s dominance over women in the same ways as culturally hegemonic forms of masculinity, which currently emphasize aspects such as prioritizing one’s physical appearance and dominating women sexually (Connell, 1995; Hoebanx, 2024). In relation to cultural events, dominant masculinity is conceptually useful for understanding how certain forms of masculinity become the most celebrated in a particular movement or social milieu, but do not reinforce men’s domination over women in the same ways as hegemonic masculinity.

## 5 Subordinate and hybrid masculinities

To illustrate the hierarchal nature of gender, Connell (1995) also conceptualized *subordinate masculinities* as occupying lower positions within the gender hierarchy, specifically in relation to hegemonic masculinity which remains ascendent. Subordinate masculinities are often aligned with other aspects of identity such as sexuality and race. For example, gay men and virtually all men of colour have been historically framed as subordinate to straight white men through both material and discursive practices (p. 74; see also Schippers, 2007). Additionally, men who are seen to lack aspects of hegemonic masculinity such as physical strength or sexual competence with women have historically been framed as subordinate to men who embody more aspects of hegemonic masculinity (see Larsen, 2020). Similarly, *complicit masculinities* consist of men who benefit from hegemonic masculinity without embodying most of its attributes themselves. As with subordinate masculinities, how much they benefit depends on their own place within the larger gender hierarchy, and varies along the lines of gender, race, disability, etc. (p. 76; see also Cheng, 1999; Connell and Messerschmidt, 2005). Complicit masculinities and subordinate masculinities are also not mutually exclusive, as men can embody forms of subordinate masculinity which are also grant them access to some aspects of male privilege and therefore reinforce men’s larger dominance over women. For example, “geek masculinity” is often characterized as both subordinate and complicit, as men who identify as “geeks” generally do not embody aspects of hegemonic masculinity such as physical strength and heterosexual prowess. However, “geeky” men are often depicted in popular culture as able to harass and even assault women, largely without repercussions, such as in the 1984 film *Revenge of the Nerds* and the 2010s television show *The Big Bang Theory* (see Larsen, 2020).

Related to complicit masculinities are *hybrid masculinities,* in which men embrace multiple, often conflicting characteristics of masculinity, and often femininity, to create new cultural masculinities (Bridges, 2010, 2014; Bridges and Pascoe, 2014; Lamont, 2015). According to Gruys and Muncsch (2020) gender scholars, men who adopt hybrid masculinities selectively incorporate “‘bits and pieces’ of ‘other’ masculinities into their gender performance” (p. 349). Doing so allows men to create new understandings of masculinity, which also change and develop through their relationships with women. In contrast to hegemonic masculinity, people and groups often construct hybrid masculinities in response to changing gender norms and relations. However, these hybrid formations still allow men to maintain aspects of structural privilege by adopting certain characteristics of masculinity, such as being perceived as “in charge” of the women in their lives (see Eisen and Yamashita, 2017; Vidmar, 2025). For example, Eisen and Yamashita (2017) find that many younger Western men construct “new” hybrid masculinities by presenting as “caring or being in touch with their feminine side,” in order to socially distance themselves from more “traditionally” masculine men and make themselves more appealing to young women (p. 801). However, despite allowing men to subvert certain aspects of hegemonic masculinity, even progressive forms of masculinity can be complicit in maintaining men’s subordination over women (Bridges, 2010, 2014; Bridges and Pascoe, 2014; Lamont, 2015). For example, adopting hybrid masculinities often allows younger men to “frame themselves as politically progressive” while still benefitting from the structural privileges which accompany masculinity (Gruys and Munsch, 2020, p. 349; see also Lamont, 2015).

Cultural scholarship on Burning Man also explores how male Burners adopt hybrid masculinity while attending Burns (Kaiser and Green, 2008, 2011; Barry, 2018). For example, Kaiser and Green (2008, 2011) describe how men at Nevada’s “Big Burn” create culturally-specific forms of “Burner masculinity” by wearing women’s clothing. While this practice allows men to enact gendered identities often stigmatized as “deviant” in the default world, they do little to actually challenge norms around sexualization of women (2011, p. 11), leaving the structural power of masculinity largely intact (p. 12; see also Gruys and Munsch, 2020).

## 6 Emphasized and dominant femininity

In discussing femininity’s broader relationship with masculinity, Connell and Messerschmidt (2005) describe all femininities as “formulated in tandem” with hegemonic masculinity, with *emphasized femininity* representing the most culturally-ascendent form within a society (p. 848). While emphasized femininity is instrumental in upholding hegemonic masculinity and men’s dominance over women (Connell, 1995; 2012; see also Messerschmidt, 2020b), all women, even those occupying the most culturally and structurally dominant positions within a society, collectively remain subordinate to men within all gender structures and relations (Connell, 1995; Blair and Hoskin, 2022). For this reason, this paper conceptually rejects terms such as “hegemonic femininity” insofar as they imply that certain forms of femininity essentially enjoy the same dominance as hegemonic masculinity. By extension, this also denotes that women who embody “hegemonic” femininity within a given society share comparable amounts of power to men who embody hegemonic masculinity (see Paechter, 2018; Schippers, 2007; Hamilton et al., 2019; Messerschmidt, 2019). While hegemonic masculinity and emphasized femininity represent the respective cultural and structural standard bearers of maleness and femaleness, important power discrepancies exist between the two. However, women in their multiple social roles are essential to “constructing [all types of] masculinities,” despite being subordinate to men (Connell, 1995, p. 73).

This paper also uses the term *dominant femininities*, which essentially serve the same conceptual purpose as dominant masculinities, representing the most culturally celebrated and/or legitimate forms of femininity in a given society. Unlike emphasized femininity, dominant femininities are not instrumental in maintaining hierarchal relations between men and women (Messerschmidt, 2020b; p. 24). Within the context of Lakes of Fire, dominant femininity refers to the culturally celebrated manifestation of empowered maternal femininity, which does not resemble emphasized femininity in the larger “default world.” Currently, the latter tends to emphasize more “traditional” forms of femininity and motherhood such as having large biological families, engaging in presentations of hyper femininity, and deferring to husbands as patriarchal heads of households (see Stotzer and Nelson, 2025).

## 7 Hybrid femininities

In relation to emphasized femininity, scholars have also explored how *hybrid femininities* are constructed (Ispa-Landa and Oliver, 2020; see also Dai et al., 2024). Similarly to hybrid masculinities (see Bridges and Pascoe, 2014), Ispa-Landa and Oliver (2020) define hybrid femininities as incorporating more “progressive” or feminist practices within more traditional understandings of femininity (p. 890; see also McRobbie, 2009; Budgeon, 2014). For example, modern hybrid femininities often combine more traditionally feminine presentations of women’s clothing and bodies with the high academic and professional achievement emblematic of liberal feminism (p. 891; see also Budgeon, 2014). For instance, Ispa-Landa and Oliver (2020) find that women within more liberal American sororities construct hybrid femininities which involve hyper feminine dress and bodily displays, while holding more progressive ideals around women’s professional achievement, when navigating relationships with “elite” fraternity men (p. 893). As gender hierarchies are both unstable and highly malleable, hybrid femininities are also influenced by “new configurations of women’s identity and practice” in which individuals reject certain aspects of emphasized femininity while retaining others, including within their relationships with men (Connell, 1995; pp. 849–850). For example, in recent decades, certain types of hybrid femininities have been increasingly acknowledged by younger and more progressive men, which hold the potential for new relations between men and women to emerge (p. 850; see also Bridges, 2014; Lamont, 2015; Gruys and Munsch, 2020; Wright, 2024). This suggests that gender identities and practices within the context of cultural events, discussed below, are similarly malleable in nature.

## 8 Doing gender in cultural spaces

Cultural events scholars outline how participants are required to continuously negotiate socio-cultural values, including those around gender (Markwell and Waitt, 2013; Pielichaty, 2015; Miller, 2016; Coyle and Platt, 2018; Hill and Sobande, 2019; Walters, 2018; Grabher, 2021). For example, Grabher (2021) notes how progressive “equality-themed events” centered on feminist consciousness-raising act as tools of meaning-making among participants, in the production of a larger culture of gender equality. In this literature, “progressivism” describes a political awareness of the systemic inequalities that exist between certain groups, notably men and women (Cock, 1989, p. 3–4). Characterizing cultural events as “progressive” thus denotes a focus both on creating spaces for marginalized groups and ameliorating the conditions these groups face (p. 97). Within progressive events, members must also perform the “transformative negotiation processes” required to perpetuate a culture of gender equality (p. 18). For example, Grabher (2021) notes how participants within cultural events often take on roles as “representatives” of gender egalitarian values and are responsible for reproducing gender roles through participation in the community and its activities (p. 19), much like Burners do in passing on the 10 Principles to others (Shister, 2019).

Similarly to Connell (1995), cultural scholars often frame events as containing gendered relations which simultaneously hold the potential to subvert existing practices and reinforce them (Pielichaty, 2015, p. 33; see also Hubbard, 2013; Grabher, 2021). For example, Hubbard (2013) finds that purportedly progressive student fancy-dress events such as the UK’s Carnage! reinforce traditional understandings of gender, as women participants often conform to ideals of emphasized femininity by displaying sexual passivity in front of male attendees (p. 265). Additionally, Grabher (2021) describes how participants’ adoption of gender practices within cultural events can reinforce the surrounding community’s values, which are often less progressive (p. 72; see also Coyle and Platt, 2018; Rodgers, 2018).

However, as Grabher (2021) notes, events not explicitly devoted to gender equality, including Burns, are largely unexplored within gender and cultural studies as “sites for the production of gender” in which gender relations are created, negotiated and contested (p. 12). These gendered processes are also under-analyzed within the existing literature on Burns more specifically (see Kaiser and Green, 2008, 2011; Chen, 2009, 2012; St John, 2019, 2017) and would benefit from greater exploration.

## 9 Data collection and methods

### 9.1 Reflexivity and research positionality

After attending the Lakes of Fire Burn in summer 2019, I became interested in understanding how its participants created and negotiated culturally-specific definitions of gender. To investigate these questions, I took on the role of what Banks (1998) refers to as an “external insider:” someone who is familiar with and appreciates the community’s beliefs, values and customs, but who was raised or socialized outside of those beliefs (p. 8). Since Burns are temporary events and no individuals have been raised primarily as Burners, I claim my external insider status from being friends with several Burners, and participating at the Lakes of Fire regional Burn. I am also currently a member of the Lakes of Fire closed Facebook group, where members discuss issues related to both Lakes and the Burner community more generally. My positionality therefore enabled me to develop a positive rapport with participants as a trusted “external insider” with a good-faith interest in understanding the community. This likely resulted in me being received differently than someone without a previous understanding of Burner values and customs. Simultaneously however, the 2019 Lakes of Fire was also the first and, to date, only Burn I had attended. This gave me a marked distance from the Lakes “regulars” who comprised my participants. Had I been a long-time Burner and knew my research participants for a similar amount of time, I likely would have introduced significant forms of bias to the interviews.

### 9.2 Population and demographics

This analysis employs semi-structured interviews with Burners who are ongoing participants in Michigan’s Lakes of Fire regional Burn. Because I focus on how gender identity structures emotional labour, gender constituted my main criteria of selection. I also selected for participants who had attended at least two Lakes of Fire Burns in the past 3 years, to ensure they were currently active in the community. To recruit, I posted two different messages in the Lakes of Fire Facebook group, stating that I was interested in interviewing Burners who have done leadership work as theme camps leaders or rangers, who patrol Lakes’ perimeter and ensure Burners’ safety. While Burners in leadership positions are overrepresented within the sample, I felt this was reasonable because these participants tended to be the most engaged in the community. Additionally, since I lived in southern Ontario and most participants lived in the American Midwest, I used this type of sampling frame to reach multiple Burners from a geographic distance. Recruitment lasted from October 2022 to March 2023. Due to geographic constraints caused by living in a different country, all interviews took place remotely. While I held most interviews over the virtual conference function Zoom, some also took place over the phone. These phone-based interviews were done at the participants’ requests, and with their permission were recorded using a phone-based app. To further protect participants’ privacy, I also destroyed the interview recordings after I completed the interview transcriptions and data analysis.

In total, I conducted 42 interviews with female (*n* = 21) and male (*n* = 21) Burners. Regarding geographic location, most participants resided in the American Midwest, (*n* = 40), with approximately half living in Michigan (*n* = 22), with the rest living in Illinois (*n* = 10) and Ohio (*n* = 10). Racially, the interview population consisted of predominantly white Burners (*n* = 39), while two male Burners identified as Latino, and one identified as African-American or Black. While this sample is largely racially homogenous, it is reflective of Lakes’ demographic make-up, as a majority white, middle-class space (Chen, 2009). In contrast, age ranges were much more diverse, and Burners varied from being in their early 20s to late 60s, with a median age of 38 years. All participants also identified as cis-gender.

I also determined socioeconomic status by taking into account factors such as participants’ jobs and, if they disclosed them, their salaries, outside lifestyle, and housing or living situations. From these, I classified participants as either working or middle/upper-middle class, depending on participants’ responses. More participants identified as middle or upper-middle class (*n* = 31) than working class (*n* = 11), which was in keeping with the larger perception of Burns as more privileged spaces largely attended by white, middle-class participants. Participants’ understandings of masculinity and femininity are also likely shaped by both race and socioeconomic status. For example, white middle-class femininity typically denotes that women display kindness, empathy and engage more in “helping” behaviours (McGinn and Oh, 2017), which are also central to the creation and maintenance of Burns. White middle-class women may thus be more socially conditioned to demonstrate these behaviours than women from different socioeconomic backgrounds, or women who are racialized. Lakes’ middle-classness may also condition how men behave relationally with women, as they are more likely to be socialized to present as ostensibly caring and respectful of women in their relationships (Lamont, 2015). Age also significantly impacts how individuals embody both masculinity and femininity; for example, some women in their 50s noted being able to take on more maternal or “grandmotherly” roles, even when they did not have biological children.

This demographic information, including the different age groups, is listed in detail in the chart (see Table 1).

**Table 1:** Demographic table.

Category	Number of participants (total =42)
Age of participant	
18–25	7
26–35	11
36–45	18
46–55	2
56–65	4
Gender	
Male	21
Female	21
Non-binary/other	0
Socioeconomic status	
Working class	11
Middle or upper-middle class	31
Race	
White	39
Black/African-American	1
Latinx	2

### 9.3 Interviewing process

Interviews lasted approximately half an hour to an hour, and were divided into two main sections. The first touched on how participants became involved in Lakes, the amount of time they participated, and their artistic and/or camp-based contributions. Some of the questions included: Can you describe the work you do at Lakes? And, given that your work for Lakes is unpaid, do you ever find it difficult to make time for it outside of other commitments? I also made sure to characterize what participants were doing as actual “work” to better frame the interview responses within the context of emotional labour, without using that term specifically. Participants were next asked how their gender impacted the types of emotional labour they performed. Questions included: Do you feel there are any gendered differences in the expectations placed on men and women in terms of how much they should contribute? And, are there any aspects of your work at Lakes that could be considered feminine/masculine that you embrace or appreciate? These questions explored how gendered expectations of behaviour impacted how Burners engaged in emotional labour. To avoid influencing responses, I also structured all questions as open-ended. I then audio recorded all interviews and transcribed them using an online transcription service.

### 9.4 Data coding and analysis

I coded the interview transcripts using NVIVO 12 software, which is commonly used to analyze qualitative data. In total, I applied three rounds of coding, each marked by a further level of analytical depth. First, I read through each transcript and wrote analytical memos, which denoted emergent themes. Next, I analyzed the interviews using abductive coding, by applying existing concepts to participants’ responses, in order to generate novel findings (see Timmermans and Tavory, 2022) and develop codes derived from central concepts from the literature. For example, I applied codes such as “Burner masculinity/femininity” by drawing both from research on gender (i.e., Connell and Messerschmidt, 2005; Gruys and Munsch, 2020) and feminist cultural events scholarship (i.e., Coyle and Platt, 2018; Grabher, 2021). In the third round of analysis, I created a series of refined subcodes which denoted specific types of Burner masculinities and femininities, giving them unique names to denote their importance to the research. I discuss these findings further in the review section below.

## 10 Findings

In this section, I first discuss the concept of “Fire Mamas,” or the maternal Burner femininities which allow women to engage in communal caretaking of others. I next describe “hybrid” or “blended” Burner masculinities, in which men borrow from both more “wholesome” forms of masculinity, while also retaining traits from more traditional but subordinate masculinities. I also discuss how forms of hyper-masculinity and hyper-femininity function as culturally-specific subordinate forms of masculinity and femininity within Lakes. Lastly, I provide an overview of the “Sacred Whore” role within Lakes, and describe how men take on parallel roles by “protecting” female victims of sexual misconduct and domestic abuse.

### 10.1 Lakes’ “fire mamas:” maternal burner femininity

At Lakes, women embraced culturally-dominant forms of hybrid femininity which combined traditional understandings of maternal femininity with progressive feminist notions of independence and empowerment. These identities allowed them to undertake emotional labour in a space which was coded as both socially and sexually progressive and deeply family-oriented, with theme camps often mirroring aspects of the traditional family. Family structures’ central role in sustaining Lakes may have been why, as women spent more time at Lakes, they often embraced new, communally-oriented identities. For example, Stevie, a camp leader, describes how being at Lakes ignited maternal feelings she had not experienced before:

There have been people who have called me Fire Mama, and I’m like, the Mama thing is weird. I’ve got a few friends [that] call me Mama Bear, and I don’t consider myself motherly. But Fire Mama [is] totally different, you know?

In embracing the maternal Burner femininity emblemized by her “Fire Mama” nickname, Stevie adopts a new maternal role which feels natural for her in the Lakes space. Lakes also encouraged women to adopt more expansive and communally oriented maternal roles, which subverted more traditional understandings of motherhood. For example, Erica, a camp leader, describes how older women like her subvert traditional understandings of mothering by embracing non-biological mothering roles:

Because I’m in the “crone zone” now, something clicked and my brain was like, I can’t have kids anymore, so I can hold babies for ladies who need that. I’m gonna be one of those little ladies who holds the grandbabies for you. We’re making the world better [if] you want [help with] babies, but I’m not the one popping out babies.

This form of maternity subverts understandings of mothering by removing the need to be a biological parent or grandparent in order to fulfill a maternal role. Erica also repurposes the “crone” stereotype, as being older allows her to tap into a form of maternal energy awakened by being at Lakes. In addition to being accessible by many women, these maternal femininities structured the emotional labour Burners expended in taking care of the larger Lakes community. For example, Ashley, a camp leader, characterizes the maternal labour she does at Lakes using relational terms:

I am a motherly person. *I’m motherly, so I inherit.* I take that on. I find ways to make people feel loved and welcome and heard, making sure the art and cooking is good.

Ashley embraces an expansive, communal form of mothering which encompasses the emotional labour needed to take care of the larger Burner community. Burners also emphasized communal forms of maternity when reflecting on the roles of others. For example, camp leader Leanna describes her campmate:

If you ever come to our camp, you’ll meet “Mama Mickey.” She is called Mama Mickey for a reason. She takes care of *all* of us.

These communal forms of maternal femininity allowed motherhood to be reimagined and reshaped by Lakes’ communal ethos, while also reinforcing more traditional understandings of women’s aptitude as caretakers and nurturers.

Communal maternal femininity also facilitated women’s ability to create substitutions for newcomers looking for alternate forms of family. For example, Annie, who leads Lakes’ “Mom Camp” comprised of “real-life” mothers, describes a situation in which she comforted a distraught young woman in need of a maternal figure:

This young lady came in, and she ended up falling apart, crying and talking about her mom, how she didn’t have a connection with her, and I was like, [come] wander with me, and talk a little bit. Some people just sense that, because we were [the] Mom Camp, people could come to us if they needed help.

In characterizing other Burners’ desire to seek out maternal figures, Annie naturalizes mothering roles by framing women as innately caring and nurturing. Moreover, in caring for individuals who seek out Lakes as an alternative to more conventional family structures, Burner women also demonstrate their commitment to Radical Inclusion, one of the 10 Principles. However, in emphasizing the importance of inclusivity within theme camps, which mirror family-based structures, Burners also naturalize women’s maternal roles as nurturers who are willing to care for anyone who comes to them.

Other Burners also characterized Michigan and the American Midwest as fostering a more matriarchal and community-minded space, which further cemented Burner maternal femininity as natural within Lakes. For example, Stevie states:

I don’t necessarily think Lakes is matriarchal, but I believe the Michigan Midwestern community is. One of the best things about [Burner] culture is it allows women to be like, I wanna drive that train or that crane, and [for men to] be like, let’s teach [you] how to fucking do it. There’s not this thing of being like, [you’re not] a dude. My boss understands the power of femininity, and that women aren’t catty, manipulative bitches.

Stevie alludes to a greater tendency among women to take on leadership roles at Lakes, and a more egalitarian form of masculinity among men in leadership; these act in tandem to allow women greater leadership roles. Some Burners also contrasted the Midwest’s more matriarchal culture to the “Big Burn” situated in the Nevada dessert. For example, Celine, a ranger, characterizes Lakes’ matriarchal ethos as unique when compared to other Burns, which are more patriarchal:

I’d attribute it to testosterone, perhaps it’s [the] traditionally [matriarchal] cultures. [Lakes is] a lot more community-centric. It’s this me, me attitude [at the Big Burn]. I’m sure, biologically, there’s probably sound attribution to masculinity.

Celine contrasts Lakes’ matriarchal communal ethos to the self-interested attitudes prevalent at the Big Burn, which she attributes to the ruggedly individualistic culture of the American West. She also states:

Things like toxic misogyny, or masculinity, I’ve seen way more of that out [at the Big Burn] than at Lakes.

Celine implies that Lakes allows women to feel more welcome than they might at other Burns. Burners who made connections between the larger Midwestern culture and more progressive forms of gender expression also often connected it to Lakes’ community-minded forms of matriarchy.

### 10.2 Sparkle Ponies: subordinate burner femininity

In contrast to the “Fire Mama” ideal, certain femininities occupied subordinate positions within Lakes, as participants framed certain Burners, known as “Sparkle Ponies,” as lazy and lacking self-sufficiency. Burners’ language when describing Sparkle Ponies was also unmistakably feminine, and included exaggerated performances of femininity characterized by both ostentatious dress, and learned helplessness (Swoffer, 2024). For example, Jordan, a ranger and camp leader, describes Sparkle Ponies as both lazy and conventionally attractive:

They’re called Sparkle Ponies, it’s the skinny festival girls who wear a bunch of glitter, get in their sexy outfits, but that’s basically their only real contribution to the camp. They don’t actually do their jobs.

Sparkle Ponies essentially embody “bimbo” roles, in which they are physically appealing but fail to contribute labour essential to sustaining Burns. In contrast to the hard-working and nurturing Fire Mamas, Sparkle Ponies inhabit a form of subordinate femininity by failing to uphold the Principles of Self-Reliance and Communal Effort. Jordan states:

They don’t do the builds, don’t tear down, don’t do maintenance. They complain, take resources, don’t bring water, are not adherent to any of the core Principles.

Within the context of Burns, Sparkle Ponies embody a form of hyper femininity which is anti-communal in nature. However, some Burners also criticized the explicitly gendered nature of the Sparkle Pony stereotype. For example, Russell, a camp leader, notes how a gendered double standard existed around Burners’ use of the term:

Let’s face it, the Sparkle Pony stereotype, of someone who doesn’t contribute, is coded as female, very coded. I imagine there’s a lot more pressure to make sure you are participating in everything you can if you are a woman, versus being male-presenting. There are tons of dudes who use excuses to be stoned for days, and no one has a derogatory nickname for them.

Russell describes how the Sparkle Pony role reinforces discrepancies between men and women around labour, as the latter are more conspicuous due to their gender and exaggerated presentations of hyper-femininity.

### 10.3 Subordinate masculinities: rejecting hyper masculinity

Generally, male Burners rejected hyper-masculinity, or forms of masculinity marked by extreme aggression, physicality, and domination (Roberts, 2022), characterizing them as undesirable and thus framing them as culturally subordinate masculinities. For example, Louis, an artist and camp leader, describes patriarchy as an oppressive ideology which sometimes infiltrates Burns:

This is still a patriarchal society, and a lot of those societal mores are ingrained. You can’t just dismiss them, you have to de-program yourself to not think that way, and it’s really difficult.

Louis characterizes patriarchal attitudes as something to be rid of, rather than an ideal foundation for masculinity or leadership. Burners also framed toxic and hypermasculinity as disruptive and unproductive. For example, Russell states:

We [always] have someone who’s a *guy* during pack-up, who decides, at that moment, [that] he is going to be the take-charge person, using the bullhorn and trying to do all their stuff, and that gets frustrating, annoys people.

In describing individuals who engage in these forms of behaviour as “guys,” Russell also masculinizes them. As Lakes relies on participants’ ongoing labour to survive, forms of toxic and hyper masculine behaviour, such as yelling and aggression toward others are inherently undesirable insofar as they are unproductive.

### 10.4 Hybrid burner masculinities and relational gender roles

In contrast to men’s rejection of hyper masculinity, Lakes’ more permissive attitude toward gender expression often encouraged them to embrace more “feminine” or otherwise gender non-conforming behaviour than they would in the “default world.” For example, ranger Anthony described how the Lakes space promotes “wholesome” forms of masculinity:

I think it’s the overall culture [that] promotes a wholesome masculinity. Whether it’s the public nudity or just acceptance [of it], the default at Lakes is to accept and welcome [others]. That’s part of the mission and the [Principle of Inclusivity].

In encouraging men to be more affectionate with one another, Lakes’ liminal nature allows them to transcend many of the wider societal taboos governing relationships with other males. In the process, gender norms around affection between men were also redefined, as the surrounding barriers dictating “acceptable” male affection dissolved. Cameron, a ranger, states:

A lot of those barriers are stripped, literally and figuratively, when you get to Lakes. Men cry and hug and are affectionate with other men, it’s the norm here.

Burners frame Lakes as a space in which societal judgments around male homosocial affection disappear and are replaced by more meaningful interactions. Lakes thus enabled new and liberating understandings of masculinity, permitting men to engage in behaviour they may have coded as overly “feminine” in the “default world,” as male Burners generally did not embrace wholesome masculinity in the same way outside of Lakes. Malcolm, a ranger, states:

I find myself acting differently here than I would in the default world, mainly because I’m much less afraid of offending people’s sensibilities.

Malcolm suggests that Lakes’ larger culture conditions men to behave in ways that they often would not when in the “default world,” in which more demonstrative forms of masculinity are less accepted.

However, male Burners also combined these “wholesome” masculinities with more analytical and managerial forms of emotional labour to create culturally dominant hybrid “Burner” masculinities which differed from the care-taking female Burners engaged in. For example, Trevor, a camp leader, engages in a masculinized form of “mothering” by doing the “left-brained” activities in his camp:

I’m sort of the “Camp Mom.” I’m the manager, so I tend to be more managerial. I’m more left-brained, I’m at the top of the org chart, [but] I try not to think of it that way. Usually, I’m helping the right-brained creative people facilitate their vision to actually get something produced.

In describing emotional labour as a form of masculine “mothering,” Trevor carves out a space for men like himself to subvert understandings of masculinity and caretaking. However, he still differentiates his role along more traditionally gendered lines, as the head “manager” at the top of the “Org Chart” who largely helps with artistic and conceptual work. His emotional labour is thus related to realizing other Burners’ artistic visions, in contrast to the caretaking emotional labour women in the camp more often engaged in. This allowed men like Trevor access to hybrid masculine identities, in which they performed gendered emotional labour which emphasized leadership and conflict resolution.

Some men also distanced themselves from femininity more explicitly by emphasizing conventionally masculine traits and qualities. For example, Thomas, a camp leader, frames his leadership style through the type of subordinate masculinity he embodied in the “default world” as an older “science guy.” Unlike Trevor however, he makes no appeals to feminine roles or characteristics in describing his work:

I am a science guy who grew up in the 1970s and 80s, I’m a product of my age. If you look at traditional masculine roles which have leadership or extraversion, those are all things that I am naturally. I don’t have a problem taking to people and groups. I can stand [in front] of 20,000 people and chat.

However, Thomas also distances himself from aspects of hegemonic masculinity:

Those are stereotypical male things, I don’t have the height, I don’t have the muscles, but I’ve got [all of] those [others].

As Thomas’s ability to embody more dominant forms of masculinity is curtailed in some ways by his physical appearance, he emphasizes other masculinities which allow him access to other forms of recognition and prestige tied to male privilege, notably those which allow him to present as an effective “leader.”

Similarly, Ron, a ranger and camp leader, describes avoiding traditional forms of “leadership” while still embodying masculine traits based in analytical and cognitive skills:

I don’t like being a leader, and I actually decline to do that a lot [of the time]. But I don’t have a problem making decisions, taking action, having linear thought processes of processing the answer. If that’s what we’re calling masculine traits, as opposed to feminine traits of intuition and feeling, I am deficient in those.

Like Thomas, Ron frames his aptitude for “leadership” as inherently masculine while explicitly distancing himself from more feminine traits. He also masculinizes these traits even when hypothetically attributing them to women:

I don’t think of those personally as male traits. I always said, I’m looking for a female me. She’s very smart, extroverted, speaks her mind. She has these *masculine* characteristics.

While characterizing his personality traits as not exclusively “male,” Ron also differentiates between the “masculine traits” he finds desirable, and qualities associated with femininity such as “intuition and feelings.”

Men also often positioned their emotional labour and masculinity relationally, contrasting them with women’s emotional labour. For example, camp leader Jamie framed his role in contrast to his female partners:

The other two co-leads of my camp are my [polyamorous female] partners, so they have a tendency to want to take care of planning meals and events. When it comes to actual interpersonal conflict, I’m usually the person who needs to have the hard conversations with people. Hard to say how much of that is gender-based or circumstantial.

Jamie demonstrates relational Burner masculinity by engaging in masculine-coded emotional labour, which compliments his partners’ more feminine labour. Despite being in a polyamorous relationship, Jamie and his female partners still retain more traditional divisions of emotional labour, while Jamie holds a more traditional role around mediating conflict. Their relationship also speaks to the potential for Burners to subvert and reimagine familial structures, while still reinforcing more traditional gender roles within them.

In contrast, when it came to differences in emotional labour, women were more likely to describe them in the context of unequal gendered discrepancies rather than natural differences. For example, Sylvia, a ranger, describes how men often engaged in more visible volunteering, while women assumed “supportive” roles:

I think men are expected to do more volunteering, and women are expected to support the men. I’ve seen many relationships [like that].

Rhea, a camp leader, also articulates how relationships between heterosexual couples often mirror traditional dynamics when it comes to emotional labour:

This is a story I’ve heard from three [different] women: We are at this Burn, I have not seen him in 24 hours, because I’m taking care of everybody else. Here I am running our camp with twenty people, and he’s the reason [why] the Burn “works.” Either he built the effigy, or is the fire safety person. I am holding down the fort, [but] he’s getting the glory as the backbone of the Burn.

Rhea suggests that Lakes’ cultural emphasis on maternal femininity serves to reconstruct more traditional relationship dynamics, particularly among couples and camp “families.” Burners often recreated the default world’s gendered dynamics around emotional labour, including the discrepancies in how much emotional labour men and women perform, and the recognition they received for it.

### 10.5 Sacred whores and male protectors

Another form of culturally dominant femininity was the “Sacred Whore” role, in which women embodied roles as sexual caretakers and educators. They were required to both educate others around sexuality and provide aftercare to survivors of sexual misconduct. Sacred Whores were thus essential in structurally facilitating consent at Lakes, which many Burners described as the “eleventh Principle.” For example, Sawyer, a camp leader, notes that Lakes allowed women like her to embrace more emboldened sexual identities focused on consent. Here she describes an experience with a woman who served as spiritual and sexual mentor:

I was in camps that were very female-positive, [like the] “House of Ill Repute.” The [leader] is a complete badass. She’s [an] Episcopalian Reverend. She didn’t just talk with people. She would spot an interesting guy, bang his brains out, and that was how I learned you could be a *Sacred Whore*. She said, I may be easy, but I am not indiscriminate, and she had such pride and centered sexuality. It was amazing to be around her, to learn from her.

In describing the Reverend as both a caregiver and sexual mentor, Sawyer juxtaposes the “sacred,” embodied in the spiritual guidance she provides to other Burners, with an empowered and agentic form of feminine sexuality. The Reverend’s status in the community also suggests that Lakes honours agency and empowerment within women’s sexuality, as her assertive sexuality does not disqualify her from being a respected mentor.

However, although the Sacred Whore role was often by Burners construed as subversive, it was also traditionally gendered, with women generally filling roles as sexual caretakers and educators. Leslie, a camp leader, connects the “sacred” nature of the Lakes space to the emotional labour women disproportionately performed to protect it:

The consent team [and] the spiritual areas of our event are almost 100% women, so I would say more women are stepping into those roles and saying, we need to hold the sacred space, we need to take care of people’s emotions. It’s really women being advocates for emotion.

Leslie characterizes sexual caretaking as inherently feminine, which men often decline to engage in. Some Burners also noted a discrepancy in the amounts of reverence men and women had for consent as an unofficial Burner “Principle.” For example, Leanna, a ranger, states:

When it comes to the sacred space, I think men should be able to hold that kind of space. Unfortunately, there are enough experiences where people have been victimized [by men] that it makes people afraid. They [have] very good reason for being afraid.

This discrepancy demonstrates the limitations of Lakes Burners to transcend more traditional gendered relations marked by misogyny, despite a culture-wide emphasis on sexual consent. Liz, a camp leader and ranger, similarly describes how education around sexual consent is still largely the domain of women, due to men’s inability, and sometimes unwillingness, to engage in sexual education and caretaking:

It has fallen on the women, and it is very bad conversation. A lot of men still minimize the impact of those behaviours, they create an unsafe environment for women. People want to be supportive of women, but because they lack understanding of what it’s like to feel unsafe as a woman, they minimize those behaviours.

Liz suggests that the differences in lived experience between men and women mean the former are often unable to be caretakers around sexuality in the same ways women are. Some men also described similar sentiments sexual caretaking. For example, camp leader Trevor notes:

I definitely see women doing it, which was good because it allows [that] someone should be, theoretically, the same gender as whoever you’re bumping into, because of those [sexual] dynamics. But to the extent that straight guys are the majority of the problem, I don’t feel [they are] doing the majority of the policing.

While empathetic towards women’s experiences, Trevor also views women as having inherently different roles in maintaining a culture of sexual consent and safety at Lakes. Curtis, a ranger, similarly notes:

[While] there are some very fluffy, cuddly ranger men I know, sadly it comes down to statistics, and most women have been sexually [abused] by men, and that’s something to be incredibly careful about, especially if they’re in a vulnerable position. Because of that, the nurturing roles tend to fall on women, who people are more comfortable with.

Curtis suggests that discrepancies in emotional labour between men and women around sexual misconduct are largely due to Lakes’ gender dynamics mirroring the default world’s, where men disproportionately harm and traumatize women. These dynamics are likely why a masculine equivalent of the Sacred Whore role is generally unavailable for men, even though Burners of all genders engage in alternative sexual practices such as BDSM and polyamory.

However, similarly to their ability to engage in “masculine motherhood,” male Burners had access to certain masculinized forms of sexual caretaking. These roles were both highly relational and traditional, as men described enforcing rules around consent and confronting perpetrators, which contrasted with women’s caretaking labour. For example, ranger Stephen states:

[If] a guy [tries] to get handsy with a woman, to the extent that we rely on women to police that, if the guy feels he has the right to cross a physical boundary with someone, he’s probably going to be more dismissive of other women trying to correct him, so I feel the obligation is on someone like myself to be at the forefront dealing with that.

Like many men, Stephen engages in emotional labour based in conflict resolution, which he frames as necessary since men are less likely to respect women’s boundaries. He also ties his role in dealing with sexual misconduct to his presentation as a conventionally masculine man:

Dealing [with misconduct] involves, sometimes, physical removal, but I don’t come across as being stereotypically feminine. A femme gay man may not be effective in curbing some guy’s behaviour, because the guy’s not going to identify with it. There’s a degree of privilege in presenting as straight.

In emphasizing his embodiment as a straight, physically imposing man, Stephen distances himself from subordinate forms of masculinity embodied by “femme gay men.” Due to his appearance, Stephen is able to access specified forms of male privilege by being physically imposing and commanding respect from other men.

Mark, a camp leader in his 60s, similarly describes engaging in masculine emotional labour, by removing men guilty of sexual misconduct. For example, when coming across an unconscious woman in an intimate position with another man:

I positioned myself in front of her and said, are you okay, even though I’ve known [the man] for years. She said no, [so] I grabbed the dude by the scruff of his neck, picked him up and said, get out of here. Now.

While Mark emphasizes his concern for the woman, his story also takes on a heroic quality:

He started to find something [to say], [but] I don’t give a fucking fuck. [I was like], get out of my face. Then I walked her [back], we found a ranger, I discussed what happened, and turned him over to the ranger. But I didn’t hesitate to do what was right, even though I knew him.

Mark frames himself as embodying a form of wholesome “chivalrous” masculinity by engaging in emotional labour based in a masculine ethic of justice, rather than a feminine ethic of care (see Gilligan, 1982), as he physically removes the perpetrator.

## 11 Discussion

Interview participants described how Burner masculinity and femininity are relational in nature within the Lakes space, and present opportunities for both subversion and reinforcement of traditional understandings of gender. For example, women enacted culturally dominant hybrid maternal femininities which borrowed from both traditional notions of motherhood, and more progressive and communal matriarchal practices. These “Fire Mama” femininities were instrumental in allowing Burners to create maternal communities not rooted in biological motherhood or the nuclear family (see O’Reilly, 2008). Women also embodied roles as respected caretakers around sexuality and consent. These femininities gave women opportunities to embrace forms of maternity many had not experienced previously in the “default world.” Scholars have broadly defined “feminist mothering” as a “counter-practice that challenge [s] the ways patriarchal motherhood constrains, regulates, and dominates women and their mothering;” this includes the notion that only biological mothers can properly care for children (O’Reilly, 2008, p. 10; see also Glenn et al., 1994). I therefore argue that maternal burner femininity also constitutes a form of “feminist mothering” which both necessitates and celebrates community and women’s independence and empowerment.

In contrast, male Burners created culturally dominant hybrid masculinities by drawing both from the gender roles they embodied in the “default world,” and the more openly affectionate forms of masculinity fostered by the Lakes of Fire space (for more on the “default world” in relation to Burns, see Shister, 2019). While male Burners rejected explicitly toxic, patriarchal and hypermasculine forms of masculinity, they also adopted more conventional, albeit sometimes subordinate masculine roles when engaging in emotional labour. For example, in roles as rangers and camp leaders, men retained aspects of the more conventionally masculine identities they held in the default world, such as those which involved having strong analytical and conflict resolution skills (for further examples of hybrid masculinities, see Eisen and Yamashita, 2017). This enabled them to maintain a certain status derived from male privilege (see Connell, 1995; Chen, 1999; Gruys and Munsch, 2020) by engaging in emotional labour involved in camp organization, conflict resolution, and art creation. Burner masculinities and femininities thus reinforce a gendered division of emotional labour which, despite Lakes’ celebration of matriarchy and progressive gender norms, recreated the more traditional dynamics of the default world in important ways.

Additionally, while some men engaged in self-described “mothering” or caretaking roles, they often downplayed the feminine nature of this work by characterizing themselves using terms like “managerial” and left-brained.” While male Burners generally had positive views of women and the work they did, most made a point of distancing themselves from overly feminine emotional labour. Burner men thus embodied complicit hybrid masculinities (see Larsen, 2020; Wojnicka, 2021), which allowed them to distance themselves more explicitly from hyper masculinity, while still granting them certain forms of male privilege, such as receiving greater recognition from others for their work. These findings reinforce scholars such as Lamont (2015) and Gruys and Munsch (2020), as many men embody hybrid masculinities which allow them to present as progressive while still benefitting from systems which reinforce gendered inequalities. Relational Burner masculinity and femininity therefore structure emotional labour in ways which ultimately reinforce gendered divisions between men and women, and keep certain aspects of men’s larger subordination of women intact.

Broadly speaking, the dominant forms of masculinity and femininity within Lakes consisted of maternal Burner femininity and wholesome but managerial Burner masculinity. While they do not enjoy the cultural ascendancy that hegemonic masculinity and emphasized femininity do in the “default world,” these forms are nonetheless instrumental in structuring participants’ emotional labour (see Messerschmidt, 2020a, b). Dominant Burner masculinities and femininities also shared specific cultural traits, notably a focus on communal behaviour and caring for others, which emphasized behaviours which evoked the Ten Principles. In contrast, Burners rejected both hyper-masculinity, which was embodied in displays of anger and toxic masculinity, and hyper-femininity, which was represented by the “Sparkle Pony” bimbo stereotype, as unproductive and therefore opposed to the Burner Principles of self-reliance and communal effort. As a result, both occupied subordinate masculinities and femininities within Lakes as a cultural milieu (see Connell, 1995; Roberts, 2022).

Spirituality also constituted a recurring theme in the interviews, particularly within the context of sexuality and women’s agency. Women often referred to Lakes as a “sacred space” whose sacredness was enjoined with consent. From this ethos the “Sacred Whore” identity emerged, which constituted a form of culturally-dominant hybrid femininity, combining a liberated understanding of sexuality with a strong ethic of maternal care. While Burning Man scholars have described how rituals are instrumental in creating cohesive community (see Gilmore, 2010; Chen, 2009, 2012), future research should explore how sexuality often informs a distinctly feminine form of spirituality in these spaces, which may also run counter to more culturally mainstream forms of spirituality within Burns.

## 12 Limitations

This paper contains limitations within its sampling, which likely impacted how Burners were able to speak to their lived experiences. For example, most participants were white, cis-gender and able-bodied; responses would likely have been different if Lakes was a majority BIPOC or LGBTQ-centred space. The article also treats emotional labour as strongly determined by the traditional gender binary. Burns have traditionally been heteronormative in nature (Kaiser and Green, 2008, 2011), and Lakes, despite being well attended by the Midwest’s queer community, is no exception. Its social structure is also predicated on familial-based camping groups, which often replicate mainstream heterosexual familial arrangements. Future research on Burns and similar cultural communities should therefore place greater focus on how queer, transgender, non-binary and other gender non-conforming participants engage with the prescribed masculine and feminine roles within these events. Notably, this research should explore how their gender identities allow them to subvert, resist or even conform to these particular gender roles (for queer-focused relational approaches to gender, see Hoskin, 2019). As queerness itself represents a subversion of established gendered roles and behaviours (Kentlyn, 2008), it is also essential to understand the ways in which queer Burners navigate these gendered structures, and how their participation has the potential to impact how cultural masculinity and femininity are reproduced and subverted.

Future studies should also interrogate how race further complicates the ways in which cultural event attendees produce and enact masculinities and femininities, particularly within spaces not explicitly devoted to promoting racial equality or certain racial groups. As Lakes remains a white-majority space, the ways in which its members enact masculinity and femininity are irrevocably shaped by their whiteness. In contrast, men and women of colour have differing degrees of access to certain masculine and feminine identities, or potentially no access at all, depending on how their racial identity interacts with aspects such as gender (for treatments of historical and contemporary non-white masculinities and femininities, see Dominiquez and Wendt, 2015; Jerald et al., 2017; Lawrence, 2020). For example, behaviour seen as “assertive” when performed by white men in leadership roles is often construed as overly aggressive when performed by Black men (Bass and Alston, 2018). Future studies should therefore focus on how not only race and gender, but also aspects of identity such as socio-economic status and disability intersect in ways which allow – or possibly disallow – participants to perform, and potentially resist, culturally-prescribed masculinities and femininities.

Additionally, in determining how representative the research findings are of the larger Lakes of Fire Burner population, it is important to acknowledge that all participants were longstanding Burners and had been attending Lakes and/or other Burns for several years. These Burners were also all in leadership roles, generally as camp leaders and/or rangers. Longer-term Burners may therefore be more likely to adopt the dominant forms of Burner masculinity and femininity, which require intensive emotional labour but also allow them to enjoy greater community recognition. It is therefore likely that Burners who attend less frequently or do not take on leadership roles also embody different forms of masculinity and femininity. While this article identifies certain forms of subordinate Burner masculinities and femininities, future research on Burns and similar cultural events should also explore how more casual attendees of these events construct different gender roles, and the purposes these serve in structuring their emotional labour.

## Data Availability

The datasets supporting the conclusion of this article will be made available by the author, upon reasonable request. Requests to access the datasets should be directed to natalie.adamyk@mail.utoronto.ca.
